# A pathway-centric view of spatial proximity in the 3D nucleome across cell lines

**DOI:** 10.1038/srep39279

**Published:** 2016-12-15

**Authors:** Hiren Karathia, Carl Kingsford, Michelle Girvan, Sridhar Hannenhalli

**Affiliations:** 1 Center for Bioinformatics and Computational Biology, University of Maryland, College Park, MD, USA; 2 Computational Biology Department, Carnegie Mellon University, Pittsburgh, PA, USA; 3 Department of Physics, University of Maryland, College Park, MD, USA.

## Abstract

In various contexts, spatially proximal genes have been shown to be functionally related. However, the extent to which spatial proximity of genes in a pathway contributes to the pathway’s context-specific activity is not known. Leveraging Hi-C data in six human cell-lines, we show that spatial proximity of genes in a pathway is highly correlated with the pathway’s context-specific expression and function. Furthermore, spatial proximity of pathway genes correlates with interactions of their protein products, and the specific pathway genes that are proximal to one another tend to occupy higher levels in the regulatory hierarchy. In addition to intra-pathway proximity, related pathways are spatially proximal to one another and housekeeping-genes tend to be proximal to several other pathways suggesting their coordinating role. Substantially extending previous works, our study reveals a pathway-centric organization of 3D-nucleome, whereby, functionally related interacting driver genes tend to be in spatial-proximity in a context-specific manner.

Recent advances in Chromosome Confirmation Capture (3C) and its high throughput derived technologies, Hi-C, have enabled genome-wide mapping of spatial proximity of genomic regions^1^,^2^,^3^. These studies emphasize the relationship between genome structure and genome function in terms of the spatio-temporal regulation of genes transcription, which are largely controlled by distal enhancer and aided by long-range DNA looping^4^,^5^,^6^. Comparative analysis of the Hi-C data across cell lines and species reveals that while the broad 3D architecture, represented by topologically associating domains (TADs) are conserved across different cell types^7^, at a finer granularity, positions of chromosomal territories, compartmentalization into active and inactive regions, and specific interactions within and across TADs vary considerably. Similarly, previous studies have shown that groups of spatially clustered enhancers exhibit co-activity across cell types and this co-activity is reflected in co-expression of spatially proximal genes, which are often functionally related^8^,^9^. More specifically, genes involved in the same pathway have been shown to be spatially proximal in *Saccharomyces cerevisiae*^10^,^11^, *Plasmodium falciparum*^12^ and *Homo sapiens* lymphoblastoid cell lines^13^. However, it is not yet clear to what extent the spatio-temporal activity of pathways is related to the spatial proximity of their constituent genes and how this relation varies in cell to cell. More generally, the broader characterization of physical proximity of genes in the context of functional pathways is incomplete and could reveal organizing principles underlying spatial proximity of pathway genes as they relate to pathway activity.

In this work, we perform a comparative pathway-centric analysis of Hi-C-derived spatial proximity data in 6 ENCODE^14^ cell types - *HEK293*^15^, *hESC*^7^, *IMR90*^16^, *BT483*^17^, *GM06990*^17^, and *RWPE1*^18^, each with replicate data. Our analysis of two large sets of pathways – KEGG^19^, and NetPath^20^ reveals several properties of spatial proximity of pathway genes. We find that, in general, the active compartment genes in a pathway tend to be significantly more spatially proximal relative to other gene pairs in the active compartment, and this tendency is even greater for gene pairs that belong to multiple pathways. While genes in spatial proximity to other genes have been shown to have higher expression, we show that this effect is especially prominent when they are spatially proximal to a gene in the same pathway. We also found that spatial proximity of pathway genes is strongly correlated with cell type-specific pathway activity. As an expected corollary, housekeeping genes, by virtue of being ubiquitously active, exhibit ubiquitous spatial proximity. Surprisingly though, we found that the protein products of spatially proximal active genes in a pathway have a significantly greater tendency to physically interact than various controls. Functional enrichment analysis suggests that spatially proximal pathway genes are enriched for specific functional classes such as RNA processing and vesicle-mediated transportation processes, and they occupy higher levels in the regulatory hierarchy within the pathway. Finally, we look at higher-level spatial organization of functional pathways by quantifying spatial proximity for all pairs of pathways. Using this, we identify a network of spatially proximal pathways that is consistent with their interdependent functional roles.

Overall, our comprehensive pathway-centric analysis of spatial proximity in multiple human cell lines shows a strong link between spatial proximity and context-specific gene expression and pathway activity. Our analysis also reveals surprising links between spatial proximity and interaction between the corresponding protein products. Functional analysis of spatially proximal genes within pathways strongly suggests a regulatory hierarchical bias in physical proximity of pathway genes. Taken together, these results are consistent with a mechanism in which early regulatory components of a pathway are brought into spatial proximity in a condition specific manner.

## Results

### Software pipeline for Hi-C processing - overview

Supplementary Fig. 1 shows the overall pipeline that, starting from the raw reads obtained from a Hi-C experiment produces significant pair-wise gene interactions. Details of the pipeline are provided in the Methods section, and we highlight a few pertinent features here. The pipeline allows the user to select a resolution at which significant interactions are identified. The pipeline uses the normalization step of the Homer tool^21^ to control for the genomic distance-dependent features of Hi-C counts (that proximal genomic regions are more likely to interact). After this normalization, the significant pair-wise interactions are not biased toward genomic proximal regions (Supplementary Fig. 2). This simplifies the sampling procedure to estimate significance of spatial proximity of a pathway (see below). Using this pipeline, we processed 6 sets of pooled replicates for 6 ENCODE cell lines - HEK293, hESC, IMR90, BT483, GM06990, and RWPE1. Supplementary Table 1 shows the data obtained for the 6 cell types at the default interaction p-value threshold of 0.001 and FDR <= 0.1. In order to assess the impact of Hi-C resolution, we independently captured the significant interactions using two different resolutions; at 10 kb and 100 kb (see Hi-C resolution dependency section in Methods).

### Spatial proximity and compartmentalization

**Figure 1:**
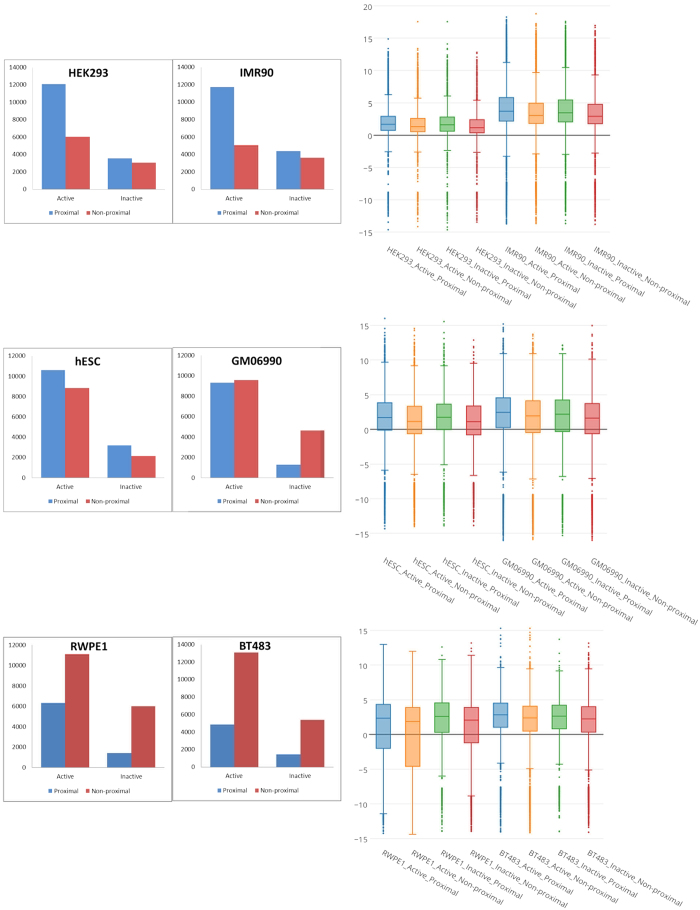
Relationships between chromatin compartments (based on HiC-PCA analysis) spatial proximity at 10 kb resolution and expression. (**A**) Genes were partitioned into active and inactive compartments based on Hi-C contact matrix as in ref. 22 and the number of spatially proximal (blue) and spatially non-proximal (red) genes in the two compartments are shown. Fisher test was performed to assess the differences in the two compartments: HEK293 (odds: 1.7; pval: 9.3e-74), IMR90 (odds: 1.9; pval: 2.2e-118), hESC (odds: 0.8, pval: 7.4e-12), GM06990 (odds: 3.5; pval: 0.0), RWPE1 (odds: 2.4; pval: 1.2e-169), BT483 (odds; 1.3; pval: 2.4e-22). (**B**) LOG2 of RNA-seq expression value (y-axis) for the genes categorized in active-proximal (AP), active-non-proximal (ANP), non-active-proximal (NAP), non-active-non-proximal (NANP) sets. Wilcoxon p-values for HEK293 (AP~ANP: 8.8e-32; AP~NAP: 1.3e-04; AP~NANP: 1.8e-46), IMR90 (AP~ANP: 8.3e-38; AP~NAP: 2.6e-06; AP~NANP: 7.2e-46), hESC (AP~ANP: 1.0e-36; AP~NAP: 0.5; AP~NANP: 3.6e-17), GM06990 (AP~ANP: 1.2e-27; AP~NAP: 5.0e-05; AP~NANP: 2.0e-41), RWPE1 (AP~ANP: 4.7e-16; NAP~AP: 3.2e-05; AP~NANP: 2.5e-05) and BT483 (AP~ANP: 1.4e-23; AP~NAP: 0.001; AP~NANP: 7.9e-25).(see Supplementary Fig. 3 for 100 kb resolution).

Previous studies have shown that the genome is divided in two compartments, called *A* and *B*, where, the *A*-type compartment is transcriptionally active and *B*-type compartment is inactive^22^. Following the pioneer work of Lieberman-Aiden^22^, we partitioned the genome into *A*-type and *B*-type compartments, independently in each cell-type using the HiC contact matrix (see method). Not surprisingly, genes in active compartments are much more likely to be spatially proximal to other genes than the genes in the inactive compartment (Fig. 1(a)), processed from the 10 kb resolution HiC contact matrix (see Supplementary Fig. 3(a) for the compartmentalization results produced through 100 kb HiC contact matrix). We further confirmed that expression is significantly higher in the *A*-compartment relative to the *B*-compartment, and within each compartment type; the spatially proximal genes have higher expression (Fig. 1(b) and Supplementary Fig. 3(b)), consistent with previous reports^23^. Given the relationships between nuclear compartmentalization, gene expression level and spatial proximity, in all of the following investigation of relationships between spatial proximity and activity of functionally related genes (as defined by pathways), we explicitly retained only the genes in active compartment (*A-compartment* or active) from each cell-line, to control for the unequal distribution of active pathway genes between the two compartments in our downstream analyses.

### Assessment of spatial proximity of pathway genes

**Figure 2:**
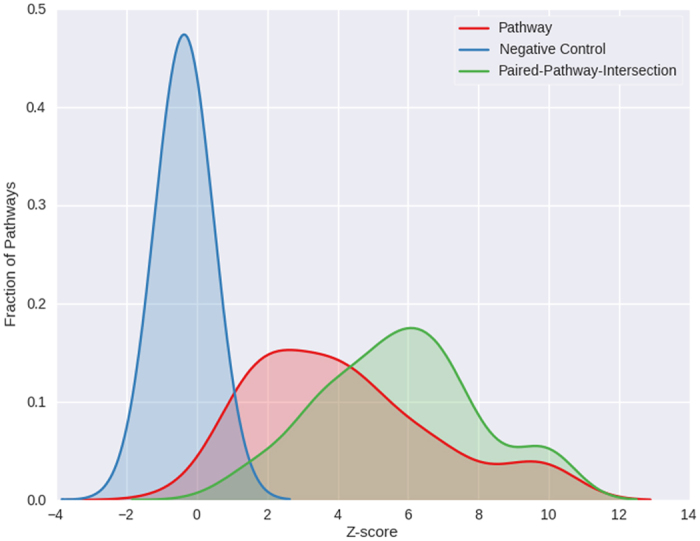
Z-score distribution of intra-pathway spatial proximity at 10 kb resolution. The figure shows the distributions of spatial-proximity z-scores for three sets of gene sets, pooled from 6 cell lines (with 10 kb resolution processed HiC data). Blue: KEGG pathway (excluding housekeeping genes). Red: Random gene-sets matching each KEGG pathway controlled for gene lengths and chromosomal distributions. Green: intersection of each pair of KEGG pathways.

We quantify cell-line-specific spatial proximity of the active genes in a pathway using *edge fraction (EF)*, which is the fraction of all possible gene pairs in the pathway that are spatially proximal (see Methods). The *EF* measure was previously shown to be effective^10^. We then quantify significance of *EF* based on a sampling procedure (see Materials & methods) and obtain a Z-score, the corresponding p-value, and multiple testing corrected q-value; higher Z-score is indicative of spatial proximity of the pathway genes above expectation. We studied 164 KEGG pathways (Release 65.0)^24^ with at least 10 genes and estimated their spatial proximity Z-scores in 6 cell lines. It is well known that Housekeeping genes show clustering along the chromosome^25^ and that may bias result in a pathways spatial proximity. To address this, we first excluded housekeeping genes from the annotated KEGG pathways and then performed the *EF* measurement and the associated downstream analyses. Figure 2 shows that overall, biological pathways tend to be spatially proximal, consistent with previous reports^10^. Interestingly, as shown in Fig. 2, we found that spatial proximity for subsets of genes shared between two pathways is even greater, suggesting that such genes, coordinating multiple pathways, is under a greater constraint to be spatial proximal.

**Figure 3:**
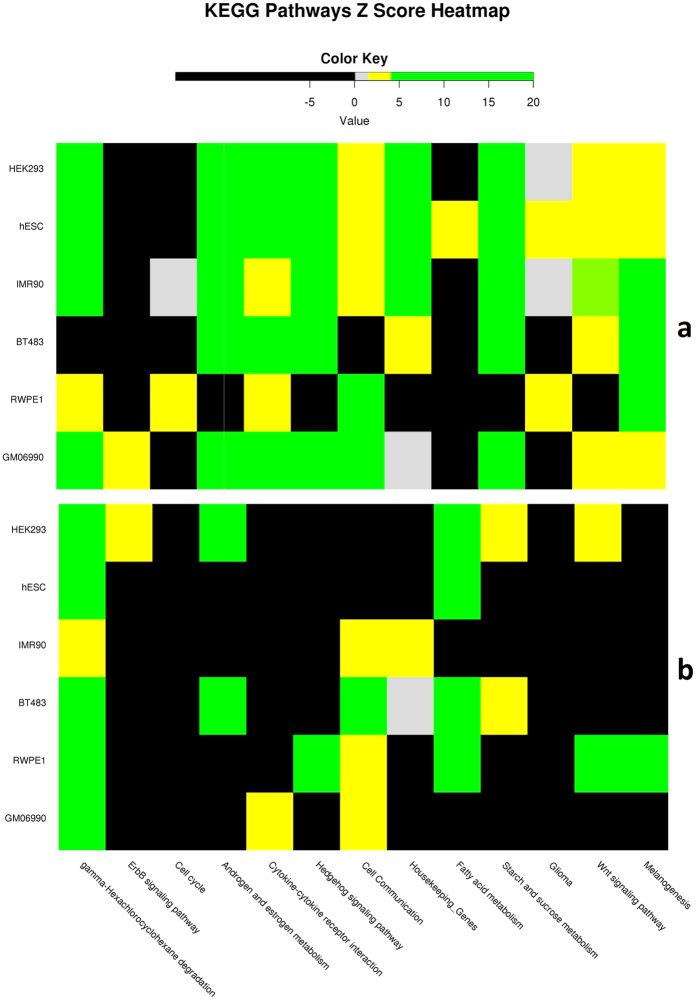
Spatial proximity of *intra-pathway* genes in selected KEGG pathways. (excluding Housekeeping genes) and Housekeeping genes, as a virtual pathway itself, across six cell lines from 10 kb (**a**) and 100 kb (**b**) Hi-C data. (Supplementary Figs 4 and 5 for full pathways).

Our primary analysis is based on processing the Hi-C data at 10 kb and 100 kb resolutions resulting in independent inference of gene-gene interaction while accounting for various confounders. Figure 3 shows the cell-type-specific Z-scores for a best representative set of pathways at 10 kb and 100 kb resolutions and Supplementary Figs 4 and 5 show the same for all pathways found spatially proximal at the respective resolutions. Consistent with the fact that the KEGG database is dominated by essential and broad cellular processes, we found that spatial proximity of KEGG pathways are not only generally high, but also a large fraction of pathways exhibits a significant level of spatial proximity in many cell types (Supplementary Fig. 6). In particular, given the ubiquitous expression and function of housekeeping genes, we tested whether these genes tend to be ubiquitously spatially proximal or whether their ubiquitous expression is decoupled from their spatial proximity. Based on 3800 housekeeping genes^26^, we first collected cell-type specific active house-keeping genes (belonging to the *A-compartment*) and estimate the *EF-Z* scores as we did for the KEGG pathways. We found that housekeeping genes exhibit significant spatial proximity to other housekeeping genes in all the 6 cell lines tested (Fig. 3 and Supplementary Figs 4 and 5). Major cancer-associated pathways (i.e., Cell cycle, Cell Communication, Wnt signaling pathway, Hedgehog signaling pathway etc.) are spatially proximal, found in many cell types perhaps suggesting a link between chromatin disruption and cancer and consistent with previous reports^27^,^28^. We found that *Cytokine-cytokine receptor interaction* pathway is spatially proximal in all the 6 cell-lines and noticeably in BT483 (Z-score = 4.3, FDR = 0.1) and RWPE1 (Z-score = 3.6, FDR = 0.1) and the interacting pair of genes in the pathway are ILR1-I-ILR1-II receptors and CXCL10-CXCL11 ligands. This lends supports to the known crucial roles of these genes in inflammation during early stage of cancer^29^. Finally, the melanogenesis pathway genes are found to be spatially proximal (at 10 kb) in all the 6 cell-lines; role of melanogenesis in prostate epithelial cells has been previously reported^30^. We also repeated pathways spatial proximity analyses with keeping only inter-chromosomal spatially proximal genes at the different resolutions and the results for the cell-type-specific Z-scores are provided in Supplementary Table 2 (see *Inter-chromosomal-proximity* worksheet).

As an additional set of pathways, we examined a set of annotated cancer-related pathways from NetPath (V.1)^20^. As shown in Supplementary Fig. 7 (10 kb and 100 kb resolutions), genes in cancer-related pathways exhibit much more subdued spatial proximity patterns in cell lines not derived from primary tumors. Mainly, we found that most Interleukin (IL) receptor induced pathways, annotated in the NetPath, are spatially proximal in IMR90 consistent with its known role in IL- Receptor induced senescence in IMR90^31^.

As a useful resource, in Supplementary Table 2 we have provided pathways in detail with underlying gene pairs that were spatially proximal in KEGG (at 10 kb, 100 kb resolutions separately), NetPath and Housekeeping gene set in different cell types. We have also provided the number of KEGG pathway genes detected in Hi-C that were also in Compartment-A as well as number of interaction between them in Supplementary Table 1.

### Spatial proximity and gene expression

**Figure 4:**
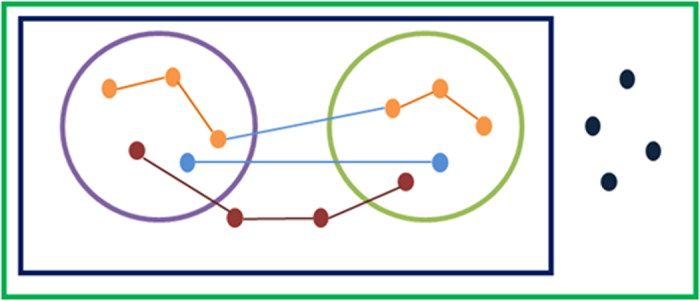
Schematic defining sets of genes and gene-pairs. Outermost rectangle represents all genes. Inner rectangle represents all genes that are in spatial proximity to at least one other gene. Circles represent annotated pathways. Nodes represent genes and edges represent spatially proximal gene pairs. All spatially proximal gene pairs are partitioned into three groups: ***proximal-intra-pathway:*** intra-pathway spatially proximal gene pairs (orange nodes and edges), ***proximal-inter-pathway:*** Spatially proximal pair of genes where each gene is in a different pathway (light blue nodes and edges), and ***proximal-generic:***Spatially proximal pair of genes such that at least one of the genes is not in any pathway (red nodes and edges). Similarly all non-proximal gene pairs are partitioned into two categories: ***non-proximal-intra-pathway:*** a non-proximal gene pair within a pathway, and ***non-proximal-generic:***any pair of genes that are not spatially proximal to any other gene and not within any pathway (pairs of dark blue nodes).

**Figure 5:**
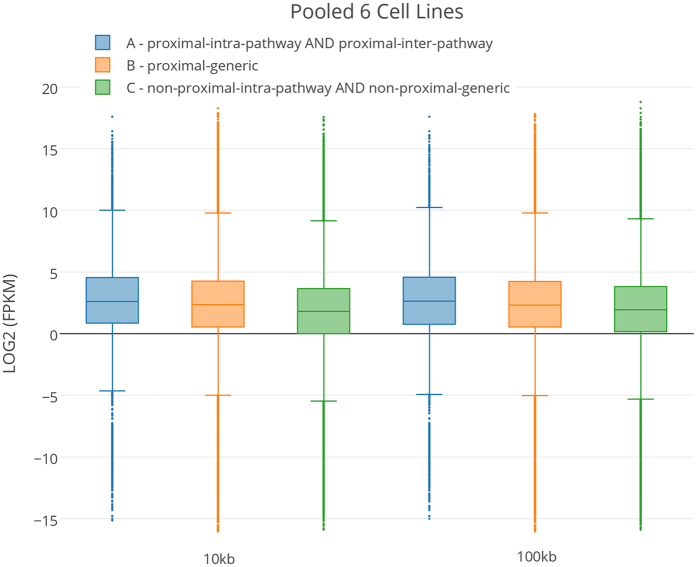
Spatial proximity and gene expression (10 kb and 100 kb resolutions). The figure shows box-plots of gene expression (FPKM) values of the genes in three different groups (see Fig. 4) pooled from all 6 cell lines. A~B Wilcoxon test 10 kb p-value = 1.5e-13 and 100 kb p-value = 1.6e-12, A~C Wilcoxon test 10 kb p-value = 1.7e-98 and 100 kb 6.8e-59. See Supplementary Fig. 8 for results of 6 individual cells at 10 kb and 100 kb resolutions.

While spatially proximal genes, largely in active compartment, are expected to have higher expression, as confirmed above, here, we assessed whether spatial proximity of genes in pathway vary across cell types in a manner consistent with their cell type-specific activity. Furthermore, we also assessed, among the spatially proximal genes, whether co-pathway membership is associated with expression level. Recall that these analyses are based only on active compartmental and highly expressed genes and are thus not biased by the known associations between chromatin compartments, spatial proximity, and gene expression. This analysis was done in all the six cell lines using RNA-seq data available in GEO (see Materials & Methods). We compared cell type specific expression levels for three disjoint groups of genes (Fig. 4). The first group consisted of genes that are spatially proximal to another gene in the pathway (*proximal-intra-pathway and proximal-inter-pathway*). The second group consisted of non-pathway genes spatially proximal to other gene (*proximal-generic*); this group was designed to assess whether shared pathway membership impinges on gene expression. The last group consisted of all other active genes not spatially proximal to any other active gene (*non-proximal-intra-pathway* and *non-proximal-generic*). Figure 5 shows that in pooled result from all 6 tissues (at 10 kb and 100 kb resolutions), while genes that are spatially proximal to other genes have a greater expression than non-proximal genes, the expression is greater for genes that are spatially proximal to a gene in the pathway. The results for individual cell lines at 10 kb and 100 kb resolutions are qualitatively similar and are shown in Supplementary Fig. 8. We also provide expression distribution of the unique KEGG pathway genes and non-pathway genes that were detected in cell specific Hi-C and were in Compartment-A (Supplementary Fig. 12).

**Figure 6:**
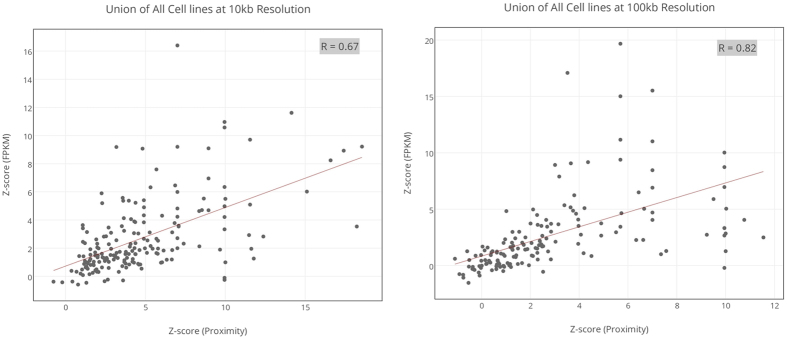
Spatial proximity versus mean pathway expression (10 kb and 100 kb resolutions). This figure shows scatter plot between proximity z-scores of pathways versus z-scores of expression values of the *proximal-intra-pathway* genes pooled from all 6 cells. Spearman rho = 0.67 (p-value: 2.7e-28) and rho = 0.82 (p-value: 6.6e-45) are respectively for 10 kb and 100 kb resolutions. See Supplementary Fig. 9 for results of 6 individual cells at 10 kb and 100 kb resolutions.

We estimated the correlation between cell type specific pathway proximity and pathway activity, approximated by the mean expression of all genes in the pathway (see Materials & Methods). The pathway activity was transformed into a Z-score, similarly to pathway proximity, based on controlled random sampling (see Methods). As shown in Fig. 6, at 10 kb and 100 kb resolution the spatial proximity is highly correlated with pathway activity (10 kb: Spearman rho = 0.67, p = 2.7e-28 and 100 kb: rho = 0.82, p = 6.6e-45). Results for all the 6 cell-lines are provided in Supplementary Fig. 9 showing the correlation of pathways proximity with its activity also high in cell specific manner. Note that these analyses do not rely on any Z-score cutoff, and is based on all Z-score for all pathways in all cell types.

### Spatial proximity and protein-protein interaction

**Figure 7:**
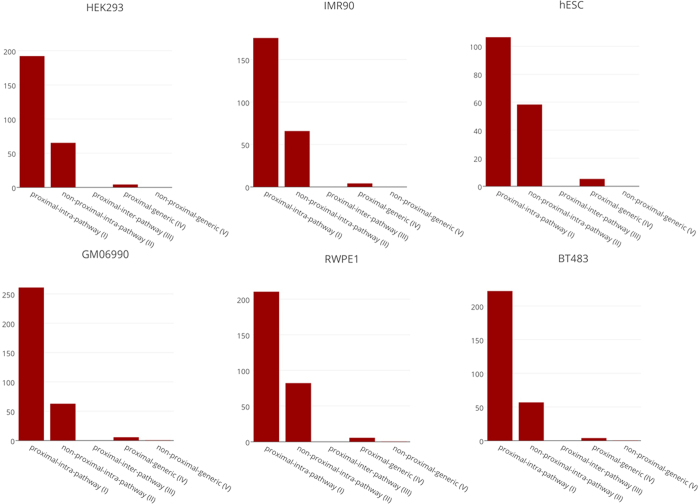
Pathway membership, spatial proximity and PPI (10 kb resolution). The figure shows fraction of gene pairs (Y-axis) in different gene groups (X-axis; see Fig. 5) whose protein products physically interact in each of the 6 cell lines. Chi-square p-value for each tissues are - HEK293 (I~II: 6.0e-04; I~III: *; I~IV: 4.0E-83; I~V: 0.0), IMR90 (I~II: 2.2e-03; I~III: *; I~IV: 1.8e-76; I~V: 4.5e-266), hESC (I~II: 0.2; I~III: *; I~IV: 8.3e-17; I~V: 2.9e-135), GM06990 (I~II: 5.1e-04; I~III: *; I~IV: 2.7e-47; I~V: 3.6e-287), RWPE1 (I~II: 0.1; I~III: *; I~IV: 8.6e-23; I~V: 1.1e-201) and BT483 (I~II: 0.2; I~III: *; I~IV: 5.9e-09; I~V: 3.5e-92). See Supplementary Fig. 10 for results of 6 individual cells at 100 kb resolutions. (*) Indicates zero fraction of PPI found in the class.

Our analyses confirms that genes in a pathway tend to be spatially proximal. Previous studies have shown that the proteins in a pathway have a greater tendency to physically interact with each other^32^. We therefore directly assessed the correlation between spatial proximity of a gene pair (independently at 10 kb and 100 kb resolution) and the physical interaction of their products. We obtained the protein-protein interactions (PPI) from HPRD database^33^ and STRING^34^ and restricted to the PPIs interactions between the *A-compartment* genes in cell specific manner. Figure 7 shows, from all the 6 cell lines, for each of the 5 groupings of gene pairs (defined in Fig. 4), the fraction of all gene pairs that have evidence for physical interaction (fractional PPI) at 10 kb resolution (results for all the 6 cell lines at 100 kb resolutions are provided in Supplementary Figs 4 and 5), which shows consistent trends in all the cell type. Taken together, these data suggest that, both pathway membership and spatial proximity of a gene pair is equally associated with PPI between their products. Furthermore, PPI tendency is much greater for gene pairs that are both in the same pathway and physically spatial proximal suggesting that the effect is not simply due to co-pathway membership. The results for 10 kb + 100 kb combined resolution data analyses also show the similar trend of proximity association with the protein-protein interactions (data not shown).

### Functional enrichment for spatially proximal genes in pathways

**Figure 8:**
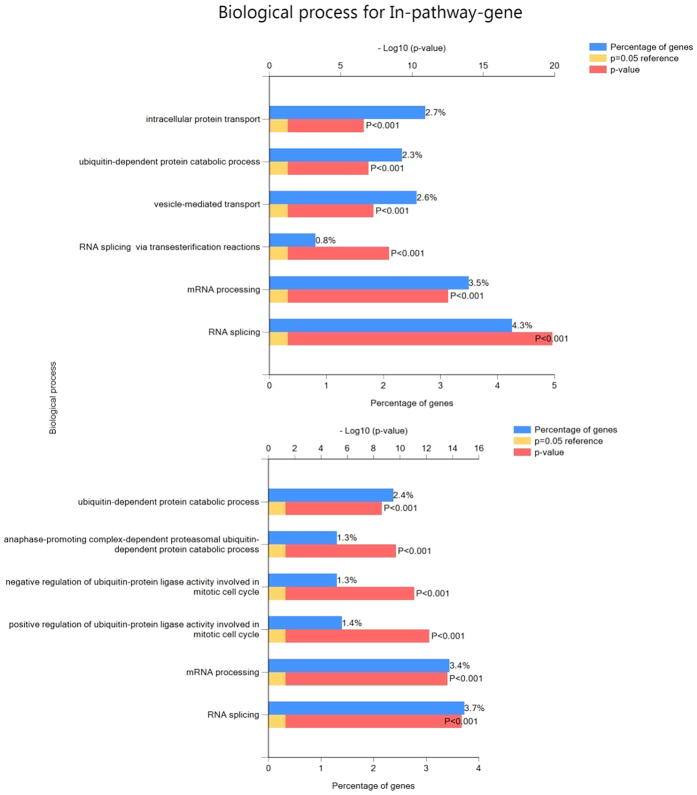
GO enrichment analysis (a. 10 kb and b. 100 kb resolution). This figure enriched different terms (GO Biological Process) at FDR <= 0.1 for active ***proximal-intra-pathway*** genes relative to other spatially proximal active genes (see Fig. 4).

Given the differences between spatially proximal and spatially non-proximal genes shown above, we investigated whether specific functional terms are enriched among *proximal-intra-pathway* genes relative to *proximal-generic* genes (see Fig. 4 for definition). We pooled the data for all pathways (at 10 kb and 100 kb resolutions independently) into two groups and performed the functional enrichment analysis using GOrilla software^35^. Figure 8 suggests that regulatory (both RNA processing, and splicing) and cellular catabolic and protein transportation processes are highly enriched among the spatially proximal pathway genes. We emphasize that our background set of genes in this analysis included those that are spatially proximal to some other gene not belonging to the pathway (*proximal-generic*). Thus, the observed functional enrichment is not due to spatial proximity alone and is a specific property of spatially proximal genes within pathways.

### Spatial proximity and regulatory hierarchy

The comparative analyses of biological properties of spatially proximal pathway genes relative to other pathway genes thus far suggests that the upstream genes in a pathway may be more likely to be spatially proximal, ensuring their robust expression and consequently robust pathway activity. We derived the hierarchical level of all pathway genes based on directed pathway edges (see Materials & Methods), and compared the hierarchical levels of spatially proximal and spatially non-proximal genes, pooled overall pathways. Our approach for assigning hierarchical level does not partition the pathway into a strict hierarchy, and can accommodate cycles. In case of 10 kb resolution, we found that, in 4 of the 6 cell lines, the hierarchical levels of spatial proximal genes were higher than the rest; Wilcoxon test p-values: IMR90 (p = 2.3E-03), GM06990 (p = 2.2E-03) and hESC (p = 4.5E-04) and RWPE1 (p = 4.4E-03). When we pool the data from the other 2 cell lines, in all the resolution cases the results are significant (i.e., p = 1.49E-09 at 10 kb). If we apply Chi-square test based on hierarchy level of 2 as the partitioning criterion, an additional cell line yields significance and importantly the odds ratio in all six cell lines range favorably from 1.17 to 5.0 analyzed in all the cell-line analyses. In case of 100 kb resolution, we found that in only 1 out of 6 cell lines, the hierarchical levels of pathway spatial proximal genes were higher than the rest (GM06990: p = 0.01). However, the range of odds ratio in all six cell-lines ranged favorably from 0.85 to 6.45.

### Spatial proximity between pathways

Next, we investigated, whether certain pairs of pathways might occupy neighboring spaces in the nucleus, suggesting their functional relatedness. Analogous to intra-pathway spatial proximity estimation, for each pair of pathways, after excluding the common genes, we obtained the number of interactions between genes across pathways and estimated its Z-score based on 1000 controlled random samplings (for computational tractability) of 2 sets of genes representing the 2 pathways. Using Z-score > 2 and FDR <= 0.1 as the thresholds, we identified a total of 1398 pathway pairs shared in at least 1 cell line, 105 of which were spatially proximal in at least 4 cell lines and 30 were spatially proximal in at least 5 cell lines (Supplementary Table 3).

Next, analogous to intra-pathway proximity analysis, we estimated spatial proximity between housekeeping genes and all other KEGG pathways. We found that housekeeping genes are significantly spatially proximal to 70 out of 164 KEGG pathways, at Z-score > 2 and FDR <= 0.1 thresholds in at least 1 cell line. The full distribution of number of housekeeping-proximal pathways shared in 1 or more cell lines is provided in Supplementary Fig. 11, and pathways spatially proximal to housekeeping genes in 4 or more and 5 or more cell lines are provided in Supplementary Table 3. These results suggest a central role housekeeping genes may play in the genome organization as well as in coordinating the activities of other pathways. Several of the pathway pairs deemed to be spatially proximal in our analysis (Supplementary Table 3) are very likely to be functionally related, for instance, pathways for signal transduction and biosynthesis thereof. Importantly, however, these data also reveal non-trivial relationships, which have some support in literature (see in the Supplementary Table 3).

Overall, these results suggest that spatial proximity between pathway genes is somewhat associated with functional interactions between the pathways.

## Discussion

In this work, we have presented a comprehensive analysis of intra- and inter-pathway spatial proximity in multiple *Homo sapiens* cell lines. Previous studies have shown that in *Saccharomyces cerevisiae*^10^, *Plasmodium falciparum*^12^ and *H. sapiens* lymphoblastoid cell line^13^, broadly, functionally related genes tend to be spatially proximal. Our goal here was to not only extend these previous observations to multiple human cell lines and assess the relationships between spatial proximity and pathway activity based on gene expression, but equally importantly, to further functionally characterize proximal genes within pathways and examine higher-order physical and functional interactions between pathways.

Previous similar analysis in *S. cerevisiae*^10^ are based on only inter-chromosomal segment interactions, and *H. sapiens* lymphoblastoid cell line results^13^ are based on low-resolution (1 Mb) segments, which can result in spurious interactions at the level of individual gene loci. Importantly, however, a greater tendency for genomically proximal regions to be spatially proximal, i.e., autocorrelation, unless appropriately controlled for, can result in false positives in inferring significant spatial proximity from Hi-C data. The absence of effective tools to control for autocorrelation has forced previous studies to exclude intra-chromosomal interactions from consideration, significantly impacting their statistical power^10^. In contrast the Homer tool^21^ satisfactorily controls of autocorrelation in estimating significance, consequently enabling us to include all significant interactions, both inter- and intra-chromosomal, in our analyses, while obviating an explicit control for inter-gene distances in random sampling procedures. We note that despite an explicit control for autocorrelation, genomically proximal regions (within 500 kb) have a slightly higher tendency to be spatially proximal (Supplementary Fig. 2). However, we explicitly tested if this biases our pathway proximity assessment as follows. We compared intra-pathway gene distances with those for randomly selected genes from the same chromosome and found that for none of the pathways there was a significant difference between the two sets of distances. Although several previous works have shown an overall tendency of co-functional genes to be genomically proximal^13^,^36^,^37^, for our filtered set of genes (those in *A-compartment* and represented in Hi-C data) do not show a systematic bias toward genomic clustering. Specifically, for instance, relative to^36^, the discrepancy may be because (1) their analysis is based on 975 genes, while ours is based on ~2500 genes, (2) they extrapolate pathways through orthology mapping across species, while we rely on human-specific annotation, (3) in their formulation they include proxy distance terms for inter-chromosomal pair of genes, which has no physical interpretation, whereas we only considered distances between intra-chromosomal gene pairs and (4) highly importantly, in random gene selection, they do not control for number of genes on each chromosome.

Tandemly duplicated genes present another potential confounding. We obtained 945 tandemly duplicated gene groups from the DGD database^38^. We found that across all cell types and Hi-C resolutions fewer than 2.5% of the tandemly duplicated gene pairs were deemed spatially proximal due to our stringent genomic proximity control. Importantly, none of our final filtered pathways included such gene pairs within a pathway. Thus excluding such tandemly duplicated gene pairs from consideration does not alter our results.

Hi-C data, like most genome-wide datasets, comes with a level of false positives, as does the pathway data. This is an important issue, and one that cannot be addressed by computational means alone. We have relied on the published interaction data that have undergone quality control measures, and used robust tool with recommended controls to perform the analyses. For pathway proximity, we have relied on a well-controlled randomized gene set to assess the significance of proximity. Despite the controls, a certain fraction of data is likely to be false positive. However, the noise in the data, as long as the tests are properly controlled, is not likely to generate strong consistent signals across multiple cell types simply by chance.

We performed a number of checks to ensure the robustness of our conclusions against several potential biases. First, note that in quantifying the significance of the pathway spatial proximity, we control for the lengths of the pathway genes. Second, we ensured that our ability to detect the spatial proximity of a pathway is independent of the number of genes in the pathway (Spearman correlation between pathway size and spatial proximity Z-score was statistically insignificant). A recent paper^9^ has suggested a link between detection of Hi-C interaction of a gene and a gene’s codon usage (which is related to its expression) and the GC content of the gene’s genomic locus. Third, we ascertained that the codon-usage does not bias the detection of Hi-C interactions (Spearman correlation = −0.09). Forth, our analyses are based only on the cell-type active genes (*A-compartment* and highly expressed genes), thus eliminating the potential biases due to differential distributions of spatial proximal genes among active and inactive compartments (Fig. 1(a and b) and Supplementary Fig. 3).

Finally, with regards to the potential GC bias, indeed highly interacting loci tend to have lower GC composition, as noted previously^11^. Although the GC composition near the restriction sites can present a technical bias^39^, several papers also suggest that GC composition may be an inherent property of the physical proximity^40^. Therefore, it seems that an explicit control for GC content may not be ideal. However, to explore the extent to which GC-controlled analyses would affect our results, for HEK293 cell line we reprocessed the data with specific GC control option provided in HOMER tool and compared the downstream results of our analyses with and without the GC control. We found that 80% of interactions detected without GC control are also detected with GC control and overall correlation between gene-wise interaction degrees between the two is 0.92. Next, we re-estimated the pathway proximity Z-scores based on GC-controlled interactions and found those to be highly correlated with the Z-score based on uncontrolled interaction detection (Spearman rho = 0.85). Lastly, we re-calculated the correlation between pathway proximity and pathway activity and found that too is as significant as the estimated without GC control (Spearman rho = 0.80, relative to 0.83 without GC control). Thus, our conclusions are not substantively biased by these various potentially confounding factors.

Our primary resource of biological pathways – KEGG, is dominated by essential and broadly utilized cellular pathways. Therefore it is encouraging to see that by and large KEGG pathways are not only highly significantly spatially proximal (Supplementary Figs 4, 5 and 6), but also a large fraction of these pathways are spatially proximal in multiple cell lines (Supplementary Table 2). However, as we show, this is not true for a different set of pathways relevant to cancer, where the overall Z-scores are much more subdued, and spatially proximity is less ubiquitous (Supplementary Fig. 7). Despite, general ubiquity of spatial proximity of KEGG pathways, we still see a strong correlation between cell type-specific spatial proximity and pathway activity as approximated by gene expression (Fig. 6 and Supplementary Fig. 9). We assessed whether our pathway proximity results are biased by shared genes among pathways. For each pathway pair, we estimated the overlap as the ratio of the intersection and the minimum of the two pathway sizes. We found that 88% of all pathway pairs have less than 5% overlap, and 92% of all pathway pairs have less than 15% overlap. Thus, pathway overlap is not of major concern in the proximity analyses.

Previous studies have noted greater transcription in spatially clustered regions^8^. Independently, earlier studies have shown the existence of so called transcription factories^41^ - nuclear locales with enriched core transcriptional machinery components where transcripts are synthesized and processed. Moreover, links between transcription factories and chromatin organization have been noted^42^. Taken together, these previous results are consistent with our observation that genes in spatially proximity with other genes have much higher expression. However, interestingly, in addition to spatial proximity alone, functional relationship between the spatially proximal genes, i.e., membership in the same pathway, makes a small but significant additional contribution to the gene expression level (Fig. 5).

Our analysis reveals an unexpected association between spatial proximity of a gene pair and the interaction between their protein products. Among the physically spatial proximal gene pairs, the well-annotated genes, i.e., annotated in some KEGG pathway, have much greater PPI propensity than genes that do not belong to an annotated pathway ((III) vs. (IV) in Fig. 7 and Supplementary Fig. 10); this may be explained by a greater representation of well-studied genes in PPI databases. Functionally related genes have been shown to have a greater propensity to physically interact^43^, consistent with our findings (compare (I) and (III) in Fig. 7). However, we found that spatial proximity is independently associated with protein interaction in both pathway ((I) versus (II) in Fig. 7) and non-pathway ((IV) versus (V) in Fig. 7) contexts. The gene-pairs that are both spatially proximal and belong to same pathway have the highest PPI propensity ((I) in Fig. 7). These trends are identical in all 6 cell lines at both the resolution – 10 kb and 100 kb (Fig. 7 and Supplementary Fig. 10), suggesting that both spatial proximity and pathway membership contribute independently to PPI propensity.

Scrutinizing each of the pathways, we found the spatially proximal genes in a pathway to have distinguishing functional characteristics relative to other pathway genes that are not spatially proximal to any other gene in the same pathway. In terms of biological processes (Fig. 8), such genes are overwhelmingly involved in transcription, splicing and intracellular transport and localization. Interestingly, the genes in a pathway that are spatially proximal to other genes in the same pathway tend to occupy a higher level in the regulatory hierarchy, related to other genes in the pathway, that also are spatially proximal to other genes but none in the same pathway. Overall, these results ascribe, for the first time, a special functional status to spatially proximal genes in pathways – such genes tend to perform higher-level regulatory functions.

We found that housekeeping genes, consistent with their ubiquitous expression and activity, tend to be broadly and highly spatially proximal. Previous studies have observed clustering of housekeeping genes into so call transcription factories^44^, and have suggested that interactions between housekeeping genes may play a role in the spatial organization of the chromatin^45^. Our results confirm these previous observations through the first genome-wide assessment of spatial proximity of housekeeping genes in multiple cell lines. In addition to spatial proximity of housekeeping genes, we also found that as a group housekeeping genes are ‘centrally’ located in the nucleus and act as a link between numerous other pathways (Supplementary Fig. 11 and Supplementary Table-3). A mechanistic interpretation of this intriguing observation, as well as of the causal link between the expression of housekeeping genes and their spatial proximity, will require further analysis.

## Conclusion

Overall, based on the first comprehensive pathway-centric analysis of spatial proximity in multiple cell lines, our results suggest that (i) context-specific regulation of pathways is associated with their context-specific spatial proximity; in doing so, our analysis provides mechanistic insights into cell type-specific activity of certain pathways, (ii) spatial-proximity of pathway genes is associated with physical interaction among their gene products, and (iii) specific classes of genes within pathways, likely occupying higher regulatory levels, have a greater tendency to be spatially proximal. Our results also provide insights into correlated activity of multiple pathways by showing that the genes in these pathways are spatially proximal.

## Material and Methods

### Hi-C processing pipeline

We downloaded paired-end Hi-C raw reads FASTQ files of sample replicates for the following tissues from GEO database (www.ncbi.nlm.nih.gov/geo): (i) HEK293 (GSM1081530, GSM1081531)^15^, (ii) IMR90 (GSM1055800, GSM1055801)^46^, (iii) hESC (GSM862723, GSM892306)^7^, (iv) GMO6990 (GSM1340639)^17^, (v) RWPE1 (GSM927076)^18^ and (vi) BT483 (GSM1340638, GSM1340637)^17^. We mapped the reads onto hg19 human genome using *BWA* tools^47^ with default parameters. The resulting SAM files were converted to BAM files using “*samtools view*” program^48^ and processed to removing PCR duplicates using “*samtools sort*” and “*Picard”* tools.

We then processed the non-redundant reads for Hi-C analysis using various HOMER tools^21^: we ran “*makeTagDirectory*” program using options “*tbp -1*” (to ensure that any genomic location is mapped by a unique read), “*–restrictionSite*” (only keep reads if both ends of the paired-end read have a restriction site within the fragment length estimate 3′ to the read), “*-removePEbg*” (removing read pairs separated by less than 1.5x the sequencing insert fragment length), “*-removeSelfLigation*” (remove re-ligation events), and “*-removeSpikes*” (remove high tag density regions).

### Normalization of Hi-C interactions

Having output from the previous steps, next we normalized the data to create individual background of the Hi-C interactions at 100 kb and 10 kb resolutions using HOMER *“analyzeHiC”* program. The program (i) divides the genome into 100 kb (or 10 kb) regions, (ii) calculates total read coverage in each region, (iii) calculates the fraction of interactions spanning any given distance with respect to read depth, (iv) optimizes a read count model to assign expected interaction counts in regions with uneven sequencing depth and (v) calculates variation in interaction frequencies as a function of distance. For fragments *i* and *j*, the procedure the estimated expected number of reads supporting the interaction as:


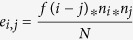


where *N* = total number of reads, *n* = number of reads in a region, and *f* represents the expected frequency of Hi-C reads as a function of distance. The enrichment was computed by taking the actual number of interactions observed between locus *i* and locus *j, m*_*ij*_, and dividing it by this expected value. The correlations are then computed as in the intra-chromosomal case, comparing the enrichment values for all inter-chromosomal locus pairs involving either *i* or *j* but excluding any intra-chromosomal locus pairs.

The background model was created for the entire data for a given sample then applied for selection of Hi-C interacting reads at default p-value cutoff of 0.001 using “*-interaction*” parameters using “*analyzeHiC*” program. We further filtered the Hi-C interactions using FDR cutoff <= 0.1 and considered the resulting set as significant Hi-C interactions. The significant Hi-C interacting reads were then passed through “*annotateInteractions*” program for mapping them onto the annotated genomic features (i.e., 5′UTR, CDS, introns, exons, intergenic etc.), from which, we selected only those interactions mapping to gene regions (i.e., Promoter, 5′ UTR, exon, intron and 3′ UTR), resulting in a comprehensive set of spatially proximal gene pairs (SGP) for each tissue.

### Hi-C Resolution dependency

In order to assess impact of HiC resolution, we captured the significant gene-gene interactions at two different resolutions: (i) at 10 kb, (ii) 100 kb. We found that the number of interactions detected was greater (i.e., in case of cell line- HEK293) when using 100 kb resolution (37206 at 10 kb versus 44977 at 100 kb). This was especially true for the inter-chromosomal interactions (297 at 10 kb versus 1538 at 100 kb). There are 8927 interactions in common between 10 kb and 100 kb resolutions. Moreover, at 10 kb resolution, large fractions of detected interactions are within a gene body, while 100 kb is a reasonable choice as it is expected to cover ~1 gene; we note that using a 100 kb resolution does not result in substantial ambiguity in gene-gene interaction mapping, as only 4% of 100 kb segments have multiple genes. Thus, to maximize statistical power with relative small fraction of ambiguous (but not necessarily biologically wrong) gene-gene interaction calls, we chose to present the results based on 100 kb resolution. However, we repeated all analyses at smaller resolution and they did not affect the conclusions; we mention these results in context.

### Separating compartmental of Genes

To classify the compartment of the human genome into *A* (active) and *B* (inactive), we adopted PCA technique, used on HiC data by Lieberman-Aiden *et al*.^22^. To perform the PCA on Hi-C data, we independently applied Homer tools ‘*runHiCpca*’ utility on normalized HiC matrix produced from 10 kb and 100 kb resolution. This utility produces a bed Graph file with positive PC1 regions, reflecting “active/permissive” chromatin, and negative PC1 regions, indicative of “inactive/inert” chromatin. Later we applied “*annotatePeaks*” utility of the Homer to quantify the PC1 values at each locus, which overlap with the genes features and consider those genes fall on positive as “active (*A-compartment*)” and on negative as “inactive (*B-compartment*)” genes. To estimate each genes expression, belonging to the A- and B-compartments, we used expression values, produced from RNA-Seq analyses (see below).

### Codon usage

To assess the effect of codon bias on interaction detection, we used Biopython’s “*SeqeUtils::ModuleCodonUsage*” package and calculated Codon Adaptation Index (CAI) for each gene of the *H. sapiens* genome.

### Pathway datasets

A ‘*pathway*’ for our purpose is a set of genes. We downloaded two sets of pathways from KEGG (release 65.0)^24^ and NetPath (V.1)^20^ databases, only retaining those with at least 10 coding genes, resulting in 164 (spanning 2545 unique genes) and 32 pathways respectively. We also included the set of 3800 housekeeping genes^26^, as an additional ‘pathway’.

### Defining classes of edges and nodes relative to pathways

For various analyses, we have defined 5 disjoint sets of genes and gene pairs in the context of a pathway and spatial proximity (see Fig. 4 for illustration). All spatially proximal gene pairs were partitioned into three groups: *proximal-intra-pathway:* intra-pathway spatially proximal gene pairs, *proximal-inter-pathway:* Spatially proximal pair of genes where each gene is in a different pathway, and *proximal-generic:* Spatially proximal pair of genes such that at least one of the genes is not in any pathway. Similarly, all non-proximal gene pairs were partitioned into two categories: *non-proximal-intra-pathway:* a non-proximal gene pair within a pathway, and *non-proximal-generic:* any pair of genes that are not spatially proximal to any other gene and not within any pathway.

### Edge fraction and its significance in estimating pathway spatial proximity

Intra-pathway gene spatial proximity was estimated as *Edge Fraction (EF)* – number of pairwise gene-gene interaction in the pathway normalized by the number of total possible interactions. To quantify significance of the *EF* for pathway with *N* genes, we randomly sampled *N* genes, such that number of genes in each chromosome is identical to real pathway, and each sampled gene’s length was within 20% of the matched pathway gene’s length (this controls for length-based bias in interaction detection). We generated 1000 such samples and calculated 1000 corresponding *EF*s. We then obtained the Z-score corresponding to the *EF* for actual pathway relative to 1000 controls. We estimated the Z-score for each non-housekeeping pathway (n = 164) and a housekeeping-gene-cluster in each cell line resulting in 165 × 6 Z-score matrix.

### Inter-pathway proximity

Analogous to the intra-pathway proximity analysis above, for a pair of pathways, after excluding the shared genes, we estimate the edges between genes in two pathways, and estimate its significance based on randomly sampling 2 gene sets (instead of 1 as above), with identical controls as above. We thus estimated a Z-score for inter-pathway proximity for all pairs of pathways.

### Processing RNA-Seq data

We downloaded raw RNA-Seq FASTQ files for three of the tissues in which matching RNA-Seq was available: (i) HEK293 (GSM1081534, GSM1081535)^15^, (ii) IMR90 (GSM1154029)^46^ and (iii) RWPE1 (GSM927074)^18^, (iv) hESC (GSM758566)^49^, (v) GM06990 (GSM958747)^50^ and (vi) BT483 (GSM1172854)^51^. The raw FASTQ reads were mapped and processed up to de-duplication steps using the same pipeline that we used to apply for Hi-C data analysis, and then used to quantify expression levels using cufflinks^52^ tool with default parameters, yielding gene-wise RPKM values.

### Significance of pathway activity

For genes with multiple transcripts, we take the maximum expression over all transcripts for the genes. For a pathway, we estimated its activity as the average expression of the genes in the pathway; we selected only the *proximal-intra-pathway* genes to estimate activity. To quantify significance of the pathway activity, we followed a sampling approach similar to that for estimating *EF* significance above, yielding a Z-score for each pathway and cell line.

### Estimating regulatory hierarchy

The regulatory analysis of a pathway requires a directed graph in which all defined interactions suggest direction of the signal flow. KEGG does not provide the directed graph by default. Therefore, we downloaded *KGML* (KEGG Markup Language), parsed the files using *KEGGgraph* package in Bioconductor, and created directed graph for each pathway using *NetworkX* package in Python. In order to investigate hierarchy of genes we first assigned a synthetic root connecting to all pathway nodes with zero in-degree, and then calculated the shortest path length (SPL) from root to every other node. Low SPL indicates higher level of hierarchy. For Chi-square and Fisher tests (see Results), we created two gene sets: (i) genes with SPL = 2 (top level) and (ii) genes with SPL >= 3 (lower hierarchy). We pooled these two sets across all pathways.

## Additional Information

**How to cite this article:** Karathia, H. *et al*. A pathway-centric view of spatial proximity in the 3D nucleome across cell lines. *Sci. Rep.* **6**, 39279; doi: 10.1038/srep39279 (2016).

**Publisher’s note:** Springer Nature remains neutral with regard to jurisdictional claims in published maps and institutional affiliations.

## Supplementary Material

Supplementary File

Supplementary Table 2

Supplementary Table 3
