# Supplementary material for: A pathway-centric view of spatial proximity in the 3D nucleome across cell lines
Source: Sci Rep. 2016 Dec 15;6:39279. doi: 10.1038/srep39279 (PMC5157015; doi:10.1038/srep39279)

SUPPLEMENTARY INFORMATION

A pathway-centric view of spatial proximity in the 3D nucleome across cell lines

Hiren Karathia[1], Carl Kingsford[2], Michelle Girvan[3], Sridhar Hannenhalli[1*]

[1]Center for Bioinformatics and Computational Biology, University of Maryland, College Park, MD

[2]Computational Biology Department, Carnegie Mellon University, Pittsburgh, PA

[3]Department of Physics, University of Maryland, College Park, MD

Authors' emails:

Hiren Karathia – hiren@umiacs.umd.edu

Carl Kingsford – carlk@cs.cmu.edu

Michelle Girvan - mgirvan@gmail.com

[*]Corresponding author

Sridhar Hannenhalli

3104G Biomolecular Sciences Building (#296)

University of Maryland, College Park, MD 20742, USA

301 405 8219 (v) 301 314 1341 (f)

sridhar@umiacs.umd.edu

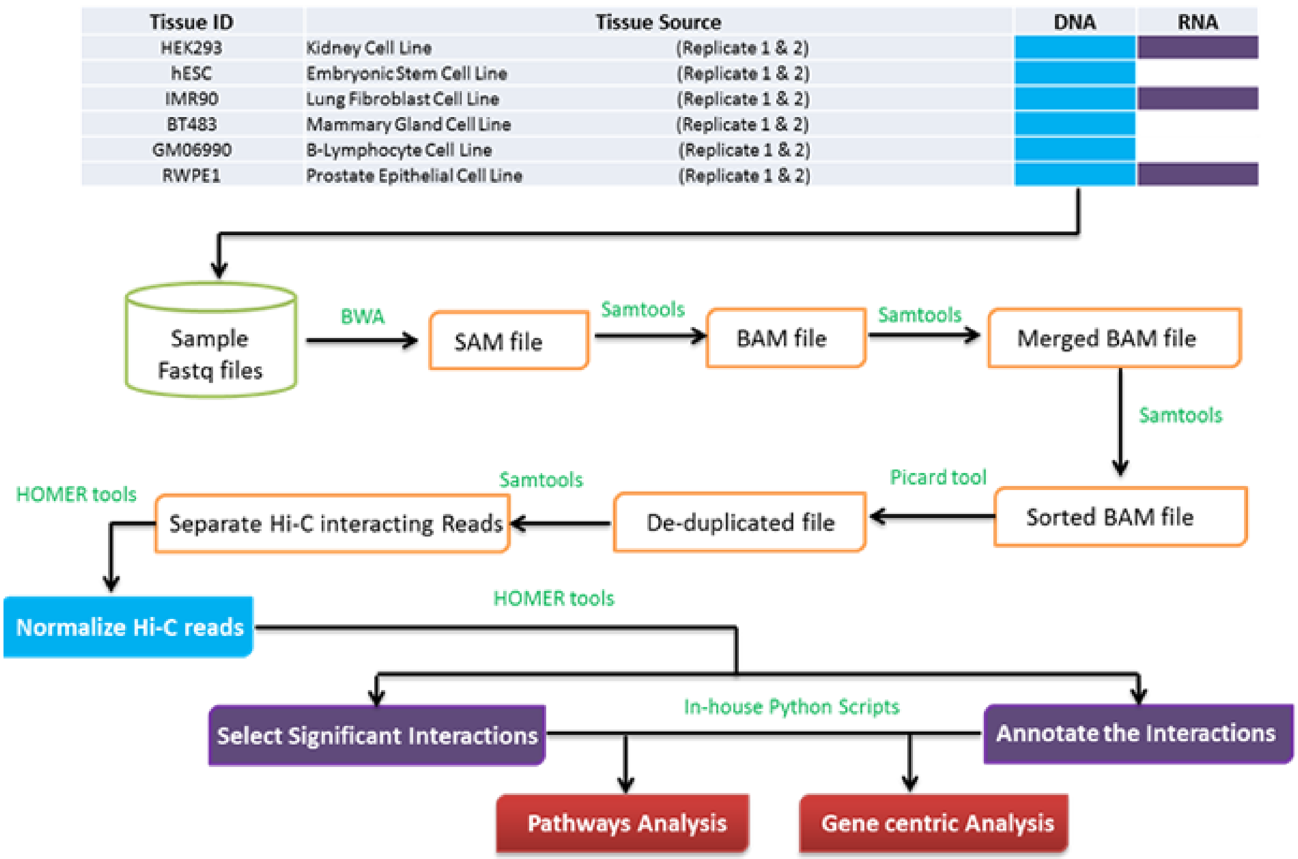

Supplementary Figure 1. Overview of the Hi-C processing pipeline and flow of downstream analysis (see Methods for details).

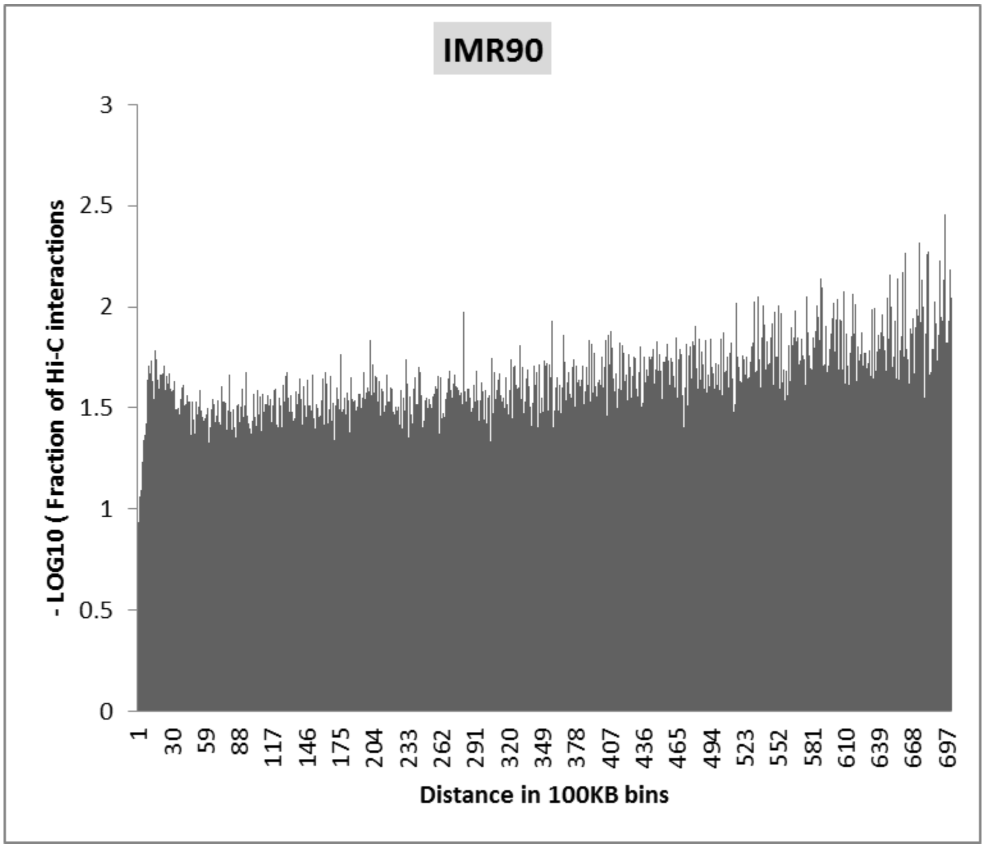

Supplementary Figure 2. Gene-gene interactions are distance independent. Y-axis shows log scaled fraction of gene-gene interactions at different inter-gene distance bins (X-axis) in IMR90.

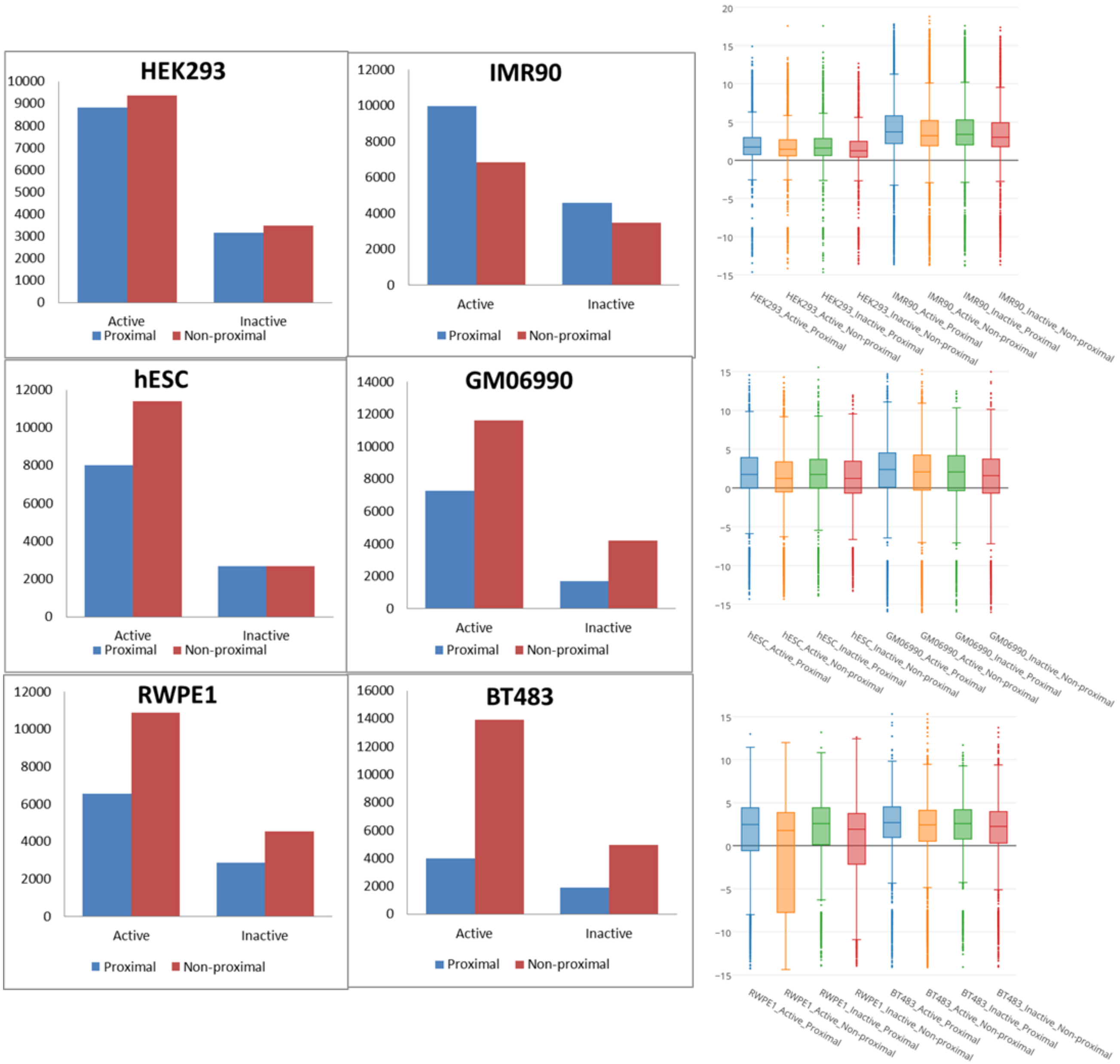

Supplementary Figure 3. Relationships between chromatin compartments (based on HiC-PCA analysis) spatial proximity at 100kb resolution and expression. (A) Genes were partitioned into active and inactive compartments based on PCA of HiC as in and the number of proximal (blue) and non-proximal (red) genes in the two compartments are shown. Fisher test was performed to assess the differences in the two compartments: HEK293 (odds: 1.04; pval:0.13), IMR90 (odds: 1.11; pval: 2.3e-04), hESC (odds: 0.7, pval: 2.5e-29), GM06990 (odds: 1.5; pval: 6.5e-41), RWPE1 (odds: 0.9; pval: 0.1), BT483 (odds; 0.7; pval: 1.2e-20). (B) LOG2 of RNA-seq expression value (y-axis) for the genes categorized in active-proximal (AP), active-non-proximal (ANP), non-active-proximal (NAP), non-active-non-proximal (NANP) sets. Wilcoxon p-values for HEK293 (AP~ANP: 1.2e-19; AP~NAP: 5.5e-05; AP~NANP: 2.2E-40), IMR90 (AP~ANP: 1.9e-22; AP~NAP: 7.7e-10; AP~NANP: 5.8E-33), hESC (AP~ANP: 3.3e-33; AP~NAP: 0.2; AP~NANP: 1.8E-16), GM06990 (AP~ANP: 8.1e-10; AP~NAP: 2.8E-05; AP~NANP: 1.5E-30), RWPE1 (AP~ANP: 7.8e-34; NAP~AP: 0.004; AP~NANP: 7.0e-17) and BT483 (AP~ANP: 0.07; AP~NAP: 0.004; AP~NANP: 3.3e-18).

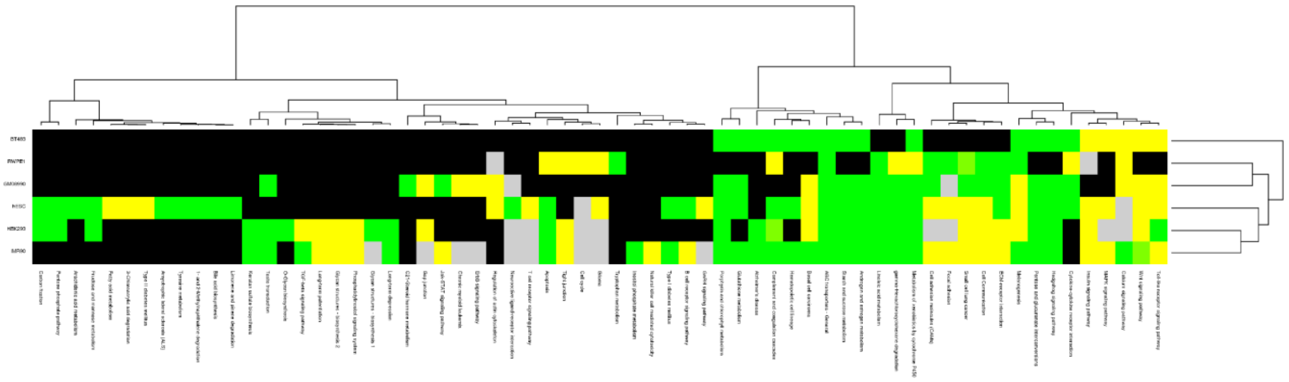

Supplementary Figure 4. Spatial proximity of *intra-pathway* genes (excluding Housekeeping genes) in KEGG pathways across six cell lines at 10kb resolution (large version is provided at the end of the supplementary information)

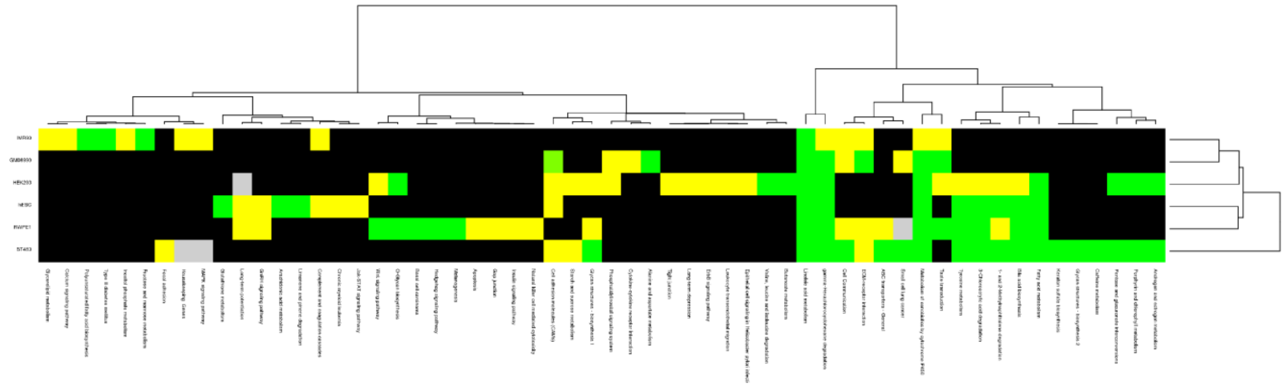

Supplementary Figure 5. Spatial proximity of *intra-pathway* genes (excluding Housekeeping genes) in KEGG pathways across six cell lines at 100kb resolution (large version is provided at the end of the supplementary information)

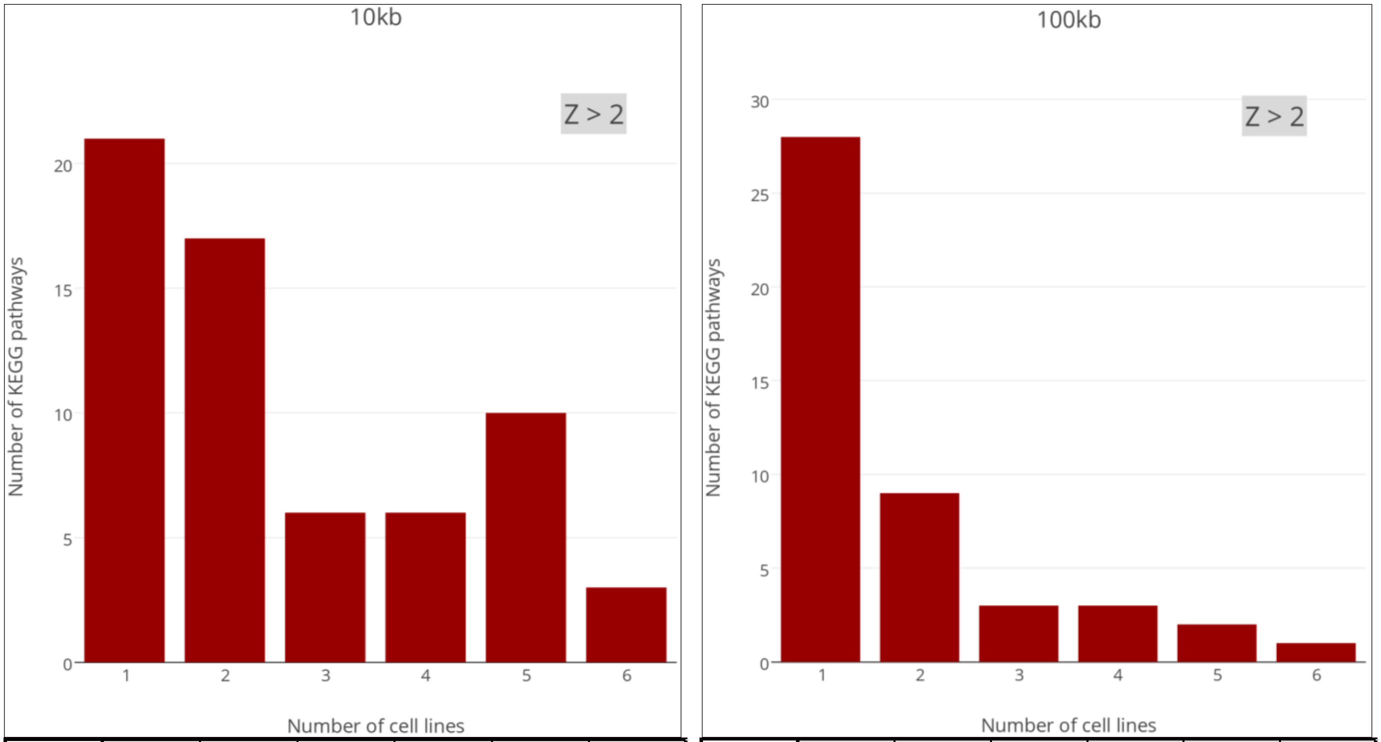

	HEK293	IMR90	hESC	GM06990	RWPE1	BT483
HEK293	11	22	16	13	19	17
IMR90		2	15	12	17	22
hESC			0	5	13	12
GM06990				2	10	8
RWPE1					4	15
BT483						2

	HEK293	IMR90	hESC	GM06990	RWPE1	BT483
HEK293	6	1	5	6	3	5
IMR90		3	1	2	2	2
hESC			8	8	7	9
GM06990				7	5	9
RWPE1					1	6
BT483						3

Supplementary Figure 6. *Intra-pathway* pathway proximity is shared across tissues. The figure shows number of pathways (without housekeeping genes) (Y-axis) with high ***intra-pathway*** proximity (Z-score > 2 and 10kb and 100kb resolutions) in different number of cell lines (X-axis). The table values show number of pathways whose ***intra-pathway*** genes proximity (Z-score > 2) is unique to a cell line (diagonal) or shared between a pair of cell lines (off-diagonal) at 10kb and 100 kb respectively.

Note: The first bar value (belonging to the unique number of pathways in a cell) is corresponding to the accumulation of the diagonal values in the table.

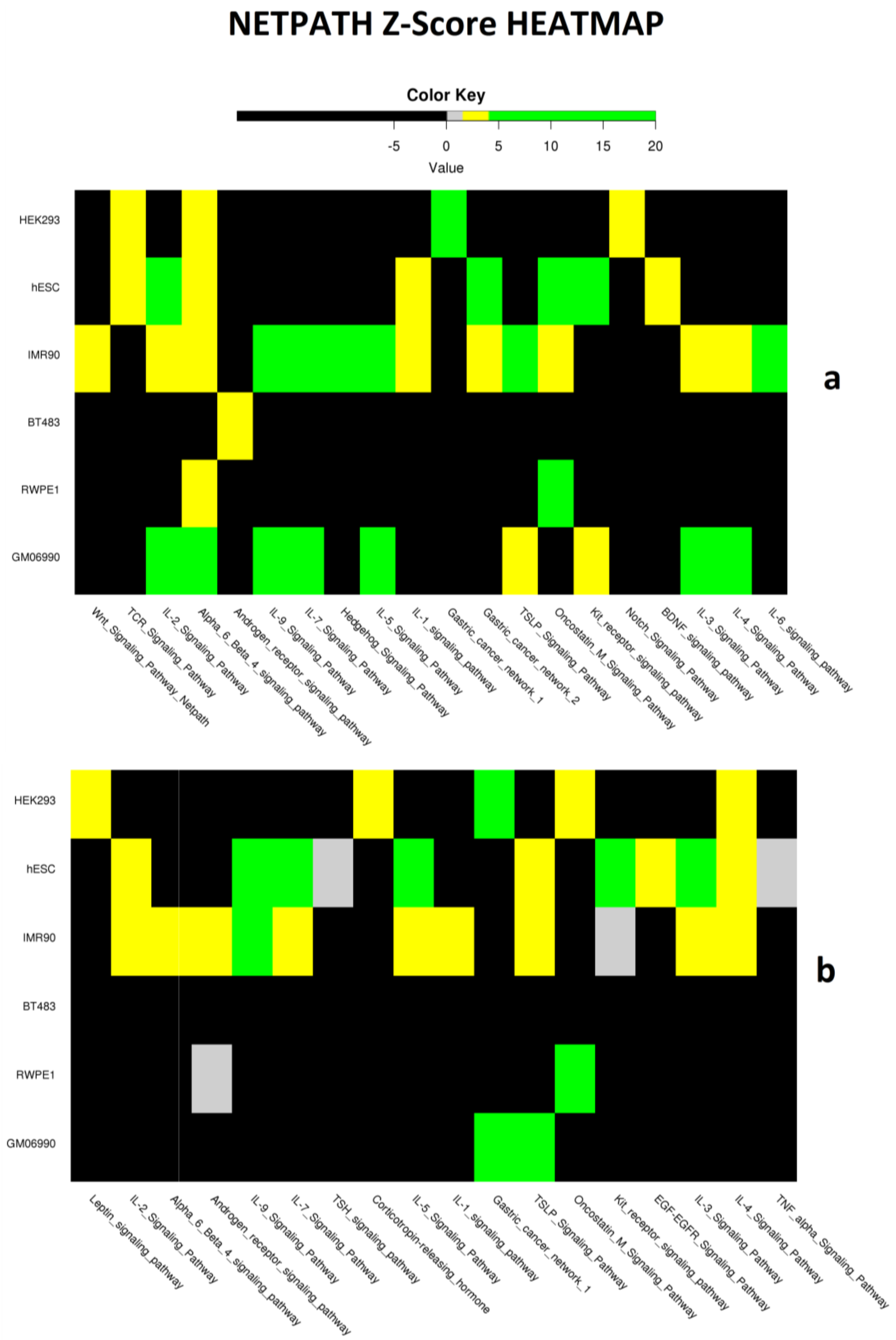

Supplementary Figure 7. Spatial proximity of NetPath annotated cancer pathways across six cell lines (a. 10kb and b. 100kb HiC processed).

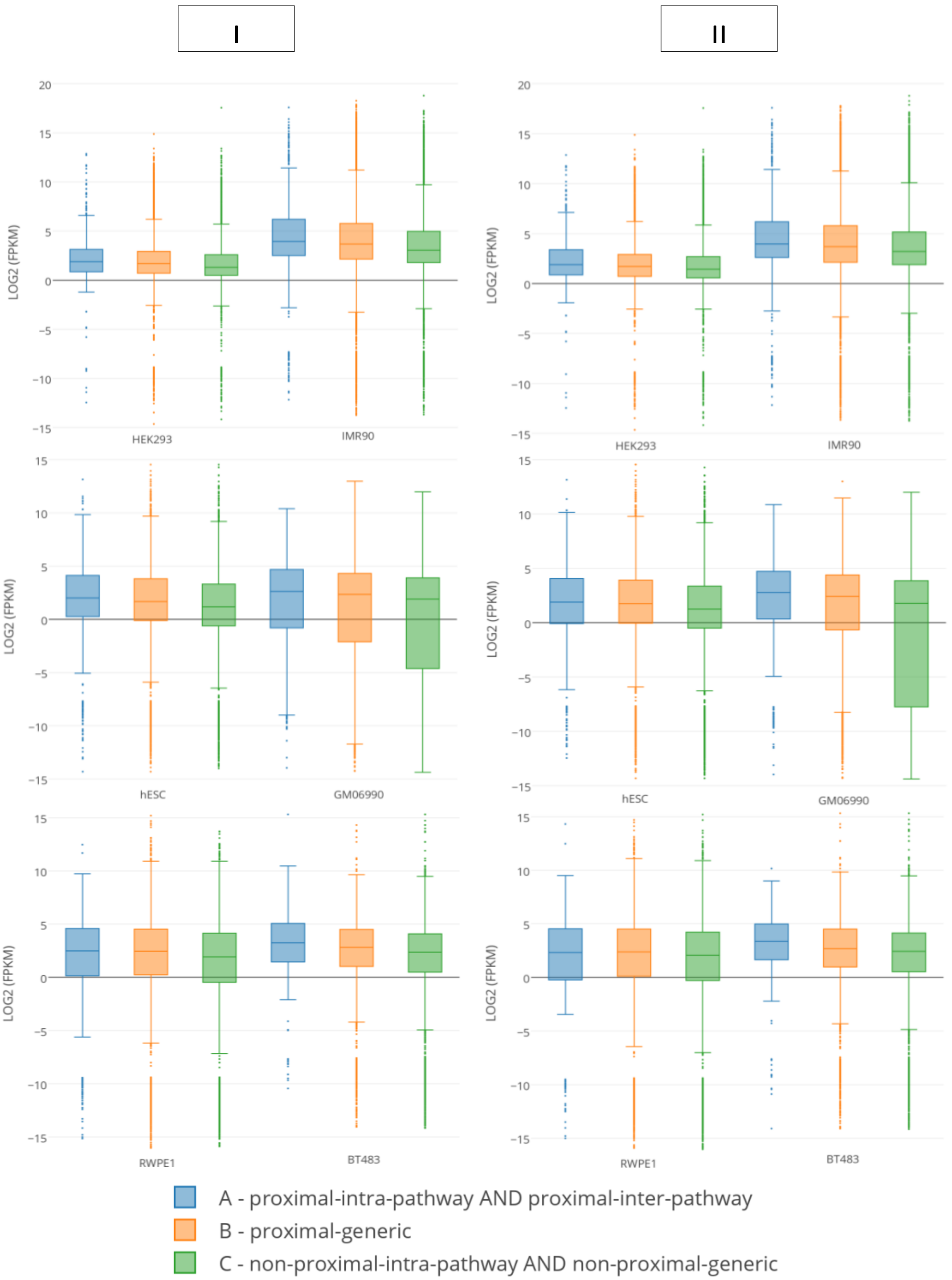

Supplementary Figure 8. Comparison of Hi-C (10kb and 100kb) genes and level of expressions. The figure shows box-plots of gene expression (FPKM) values of the genes in three different groups at (I) 10kb and (II) 100kb resolution (see Figure-4). In case of cell-lines – Wilcoxon test p-values for HEK293: A~B (I: 3.4E-03; II: 3.3e-03), A~C (I: 2.7e-13; II: 2.3e-08), IMR90: A~B (I: 5.3e-04; II: 3.4e-04), A~C (I: 3.9e; II: 1.7e-12), hESC: A~B (I: 3.7e-03; II: 0.1), A~C (I: 3.6e-12; II: 2.4e-06), GM06990: A~B (I: 0.4; II: 0.5), A~C (I: 8.3e-04; II: 0.1), RWPE1: A~B (I: 0.01; II: 0.02), A~C (I: 7.2e-06; II: 3.4e-08), BT483: A~B (I: 9.2e-03; II: 5.8e-03), A~C (I: 3.4e-06; II: 4.1e-05).

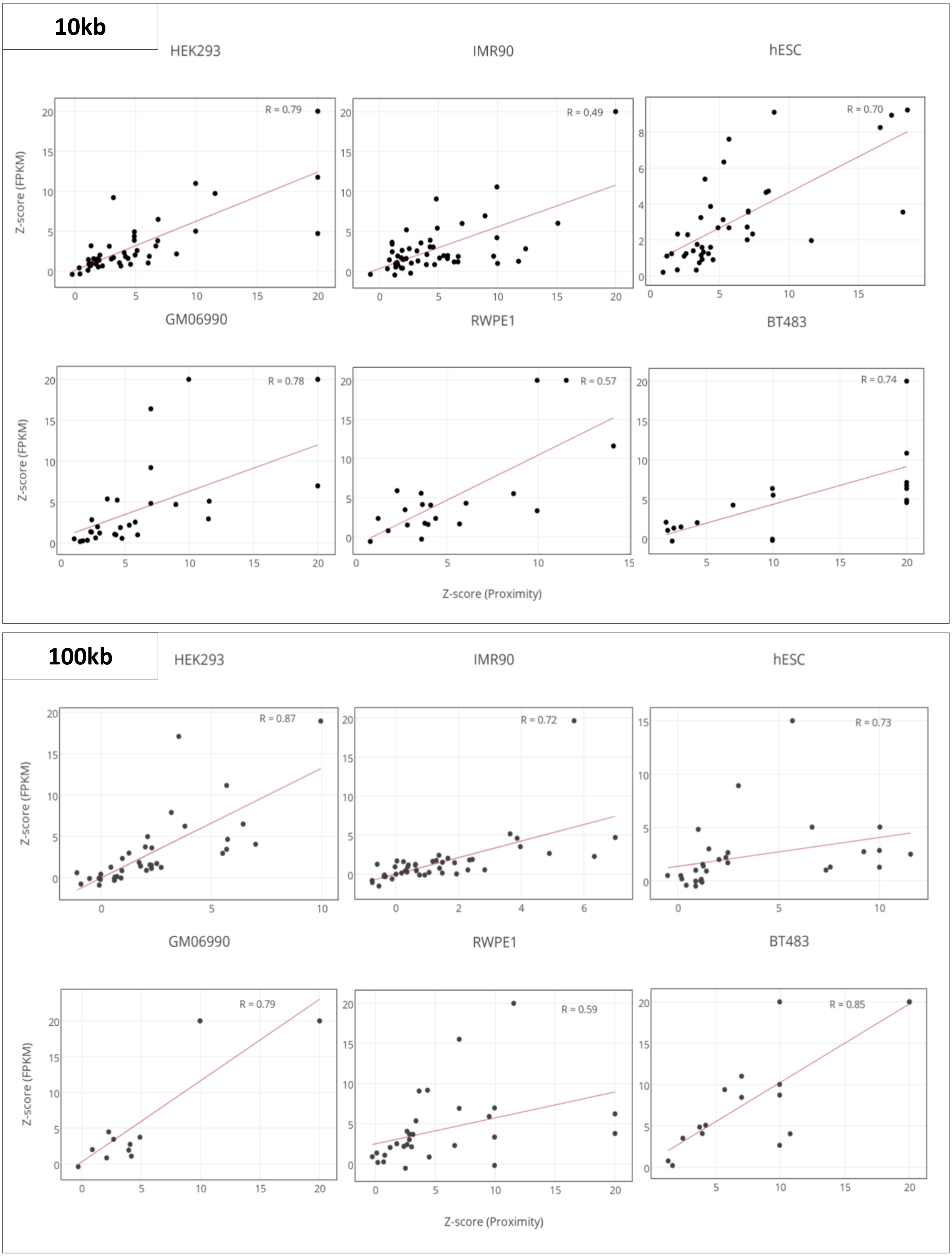

10kb
HEK293
R = 0.79
IMR90
R = 0.49
hESC
R = 0.70
GM06990
R = 0.78
RWPE1
R = 0.57
BT483
R = 0.74
Z-score (FPKM)
Z-score (Proximity)
100kb
HEK293
R = 0.87
IMR90
R = 0.72
hESC
R = 0.73
GM06990
R = 0.79
RWPE1
R = 0.59
BT483
R = 0.85
Z-score (FPKM)
Z-score (Proximity)

Supplementary Figure 9. Spatial proximity versus mean pathway expression (10kb and 100 kb resolutions) for all the 6 cell lines. This figure shows scatter plot between proximity z-scores of pathways versus z-scores of expression values of the proximal-intra-pathway genes in all the 6 cells. Spearman rho for the corresponding cell-line is displayed in the box and the respective p-values for 10kb and 100kb resolutions for cell-lines - HEK293 (7.2e-11 ; 1.1e-13), IMR90(1.0e-03 ; 3.0e-08), hESC (5.6e-08 ; 2.8e-06), GM06990 (5.9e-07 ; 1.2e-03), RWPE1 (8.4e-03 ; 7.2e-04) and BT483 (2.5e-04; 2.6e-07).

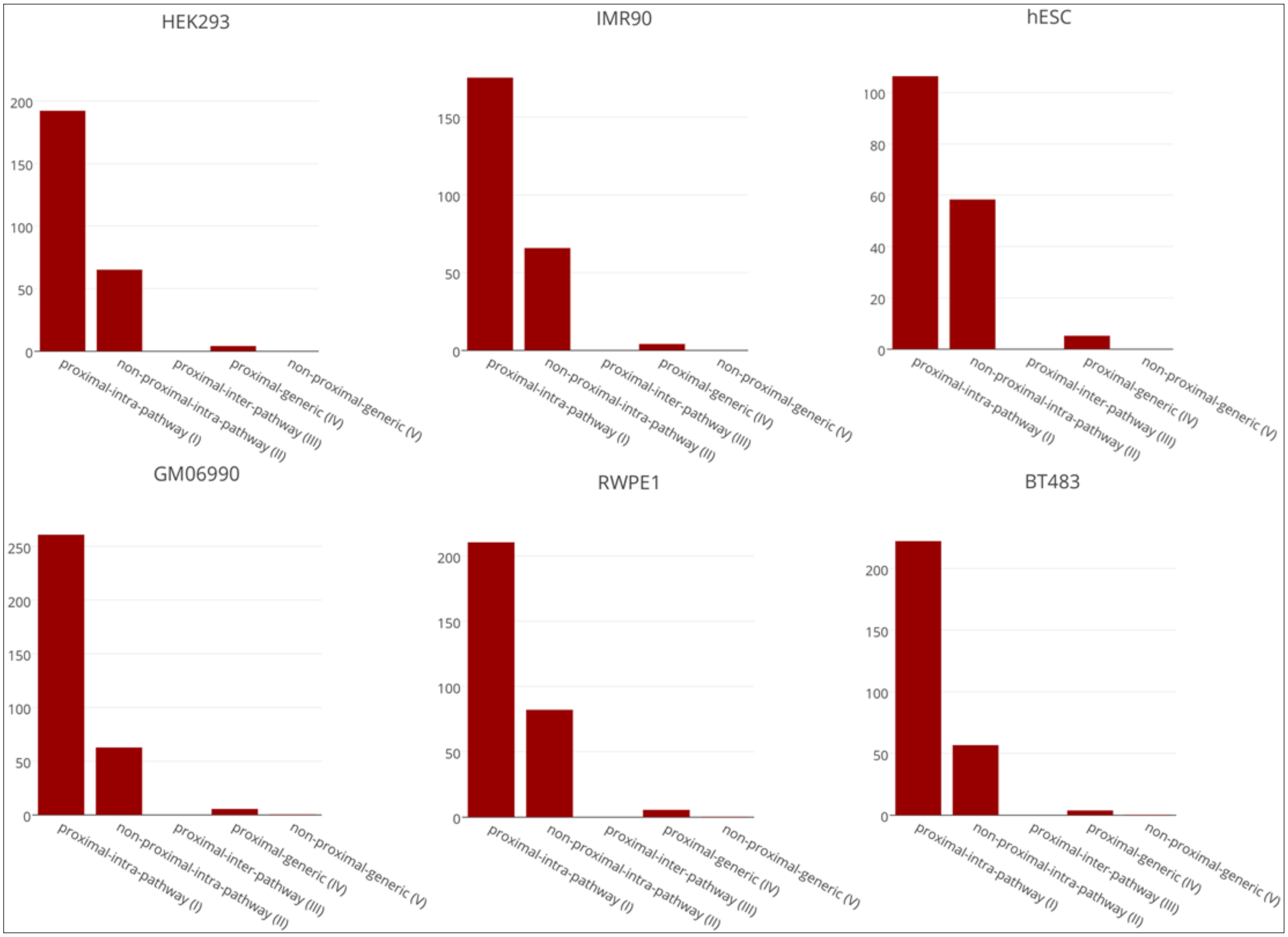

Supplementary Figure 10. Pathway membership, spatial proximity and PPI at 100kb resolution (see Fig. 4 legend). Chi-square p-value for HEK293 (I~II: 0.4; I~III: *; I~IV: 2.2e-13; I~V: 2.5e-54), IMR90 (I~II: 0.1; I~III: 8.7e-03; I~IV: 1.1e-27; I~V: 1.5e-112), hESC (I~II: 0.1; I~III: 0.05; I~IV: 2.8e-24; I~V: 2.3e-185), GM06990 (I~II: 0.7; I~III: *; I~IV: 0.01; I~V: 1.2e-16), RWPE1 (I~II: 0.01; I~III: *; I~IV: 2.5e-35; I~V: 8.4e-213) and BT483 (I~II: 0.1; I~III: *; I~IV: 3.5e-10; I~V: 3.4e-79).

(*) indicates zero fraction of PPI found in the class.

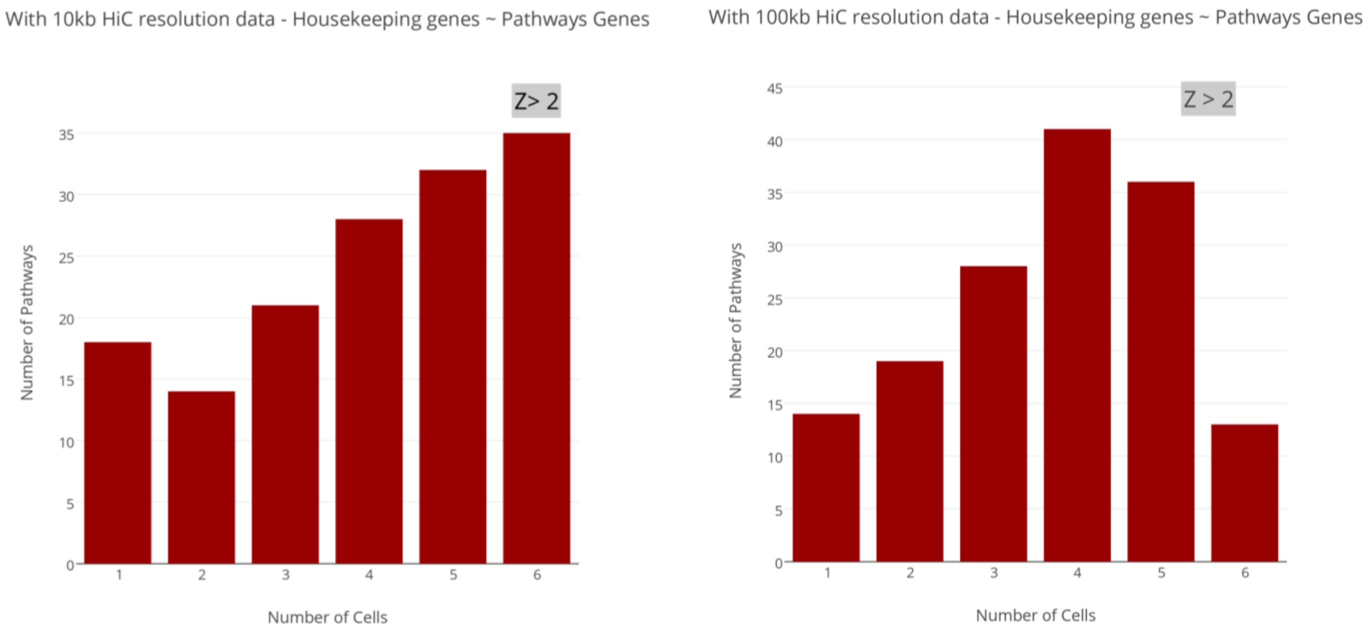

Supplementary Figure 11. Housekeeping centric proximity of KEGG pathways. This figure shows number of pathways (Y-axis) whose non-housekeeping genes are significantly proximal to the housekeeping genes at z-score > 2 (10kb and 100kb with FDR = 0.1) in different number tissues (X-axis). List of the Housekeeping-genes centric pathways are provided in Supplementary Table-3

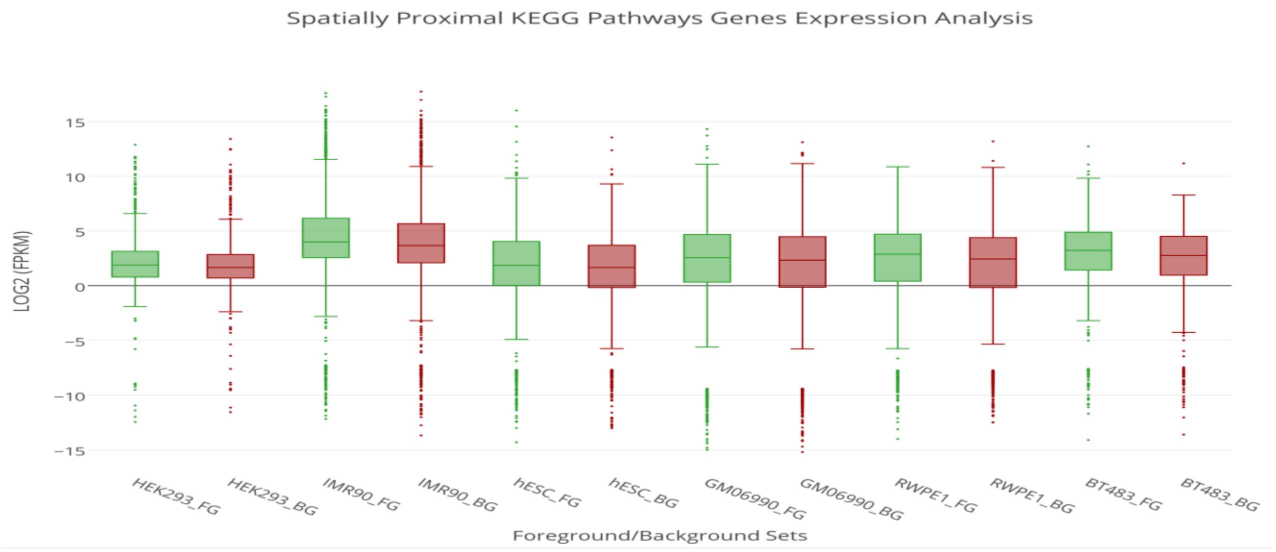

Supplementary Figure 12. Distribution of the gene expression (FPKM) for the unique KEGG pathways genes detected in HiC and Compartment-A and the same number of genes randomly chosen from the non-KEGG pathway genes detected in Hi-C and in Compartment-A on the same chromosome. P-values for the one-sided distribution difference for FPKM (KEGG-gene > Non-KEGG-Genes) (I) HEK293 (p-val: 4.8e-03) (II) IMR90 (p-val: 2.3e-05), (III) Hesc (p-val: 2.5e-02), (IV) GM06990 (p-val: 3.3e-02), (V) RWPE1 (p-val: 1.0e-02), and (VI) BT483 (p-val: 5.5e-03).

Supplementary Table 1. Hi-C analysis summary.

Categories	HEK293	IMR90	hESC	GM06990	RWPE1	BT483
Replicate-1: Hi-C mapped fragments in million	66.69	92.03	42.49	58.62	41.8	73.65
Replicate-2: Hi-C mapped fragments in million	299.86	210	234.33	96.33	64.7	82.33
# Unique HiC interactions (at 10kb)	37206	37292	18398	11440	6214	4059
# Unique genes involved in the HiC interactions (at 10kb)	15731	16143	13831	10623	7742	6335
# Unique HiC interactions in *A-compartment* (at 10kb)	27028	26618	12254	10115	5097	2920
# Unique KEGG pathway genes detected in H-C and in *A-compartment* (at 10kb)	1580	1533	1389	1223	854	600
# Unique HiC interactions in *A-compartment* among 2545 pathway genes analyzed (at 10kb)	533	541	263	204	104	55
# Unique genes involved interactions in *A-compartment* (at 10kb)	12108	11721	10596	9315	6317	4859
# Unique HiC interactions (at 100kb)	44977	101502	34531	11447	14124	5792
# Unique genes involved in the HiC interactions (at 100kb)	11978	14550	10713	9019	9416	5948
# Unique HiC interactions in *A-compartment* (at 100kb)	25300	49850	17984	8734	8008	2856
# Unique genes involved interactions in *A-compartment* (at 100kb)	8801	9958	8015	7283	6536	4008
# Unique KEGG pathway genes detected in H-C and in *A-compartment* (at 100kb)	1167	1278	1027	1013	876	541
# Unique HiC interactions in *A-compartment* among 2545 pathway genes analyzed (at 100kb)	554	1079	367	212	191	76

Supplementary Table 2. External excel sheets. Tables for all non-housekeeping KEGG and NETPATH pathways and housekeeping interactions considered, the specific gene pairs that were proximal (at 10kb and 100kb resolutions) in different cell lines.

Supplementary Table 3. External excel sheets. Tables for pathway pairs those are proximal to each other and proximal to Housekeeping genes (at 10kb resolution).

KEGG Pathways Z-scores at 10kb resolution

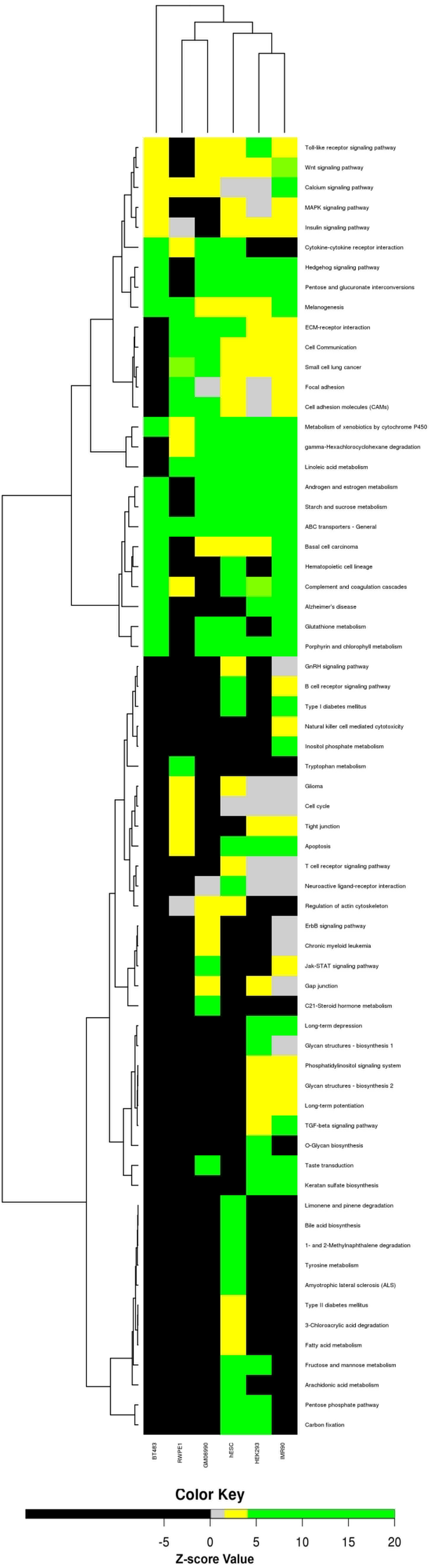

KEGG Pathways Z-Scores at 100kb Resolutions

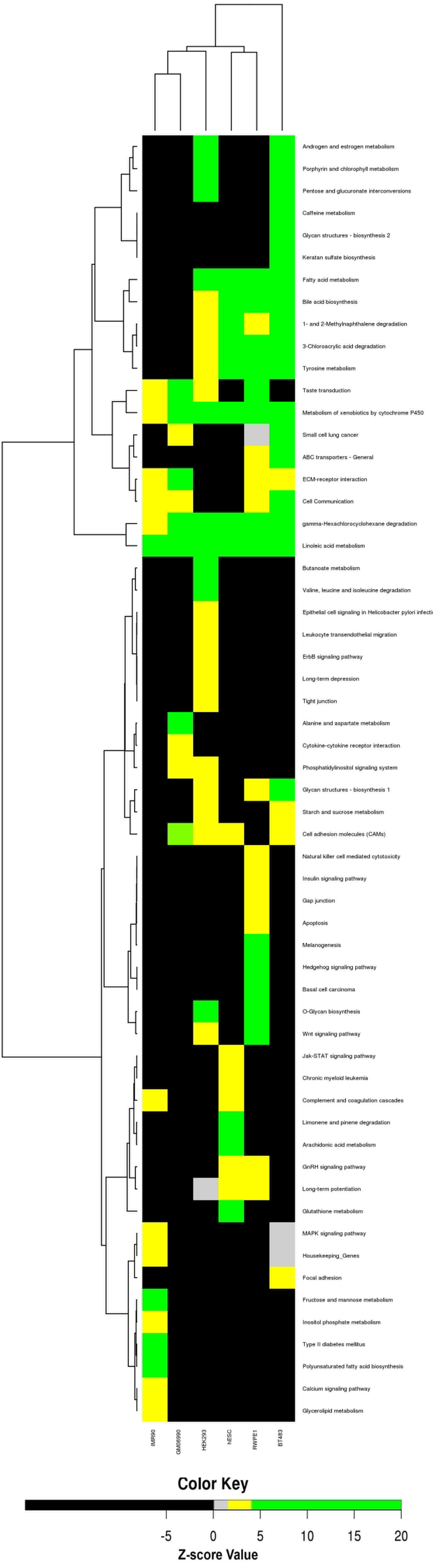

Supplement: Supplementary File [file srep39279-s1.pdf]
